# A novel *cis*-regulatory element regulates αD and αA-globin gene expression in chicken erythroid cells

**DOI:** 10.3389/fgene.2024.1384167

**Published:** 2024-04-19

**Authors:** Josué Cortés-Fernández de Lara, Hober Nelson Núñez-Martínez, Gustavo Tapia-Urzúa, Sylvia Garza-Manero, Carlos Alberto Peralta-Alvarez, Mayra Furlan-Magaril, Edgar González-Buendía, Martín Escamilla-Del-Arenal, Andrea Casasola, Georgina Guerrero, Felix Recillas-Targa

**Affiliations:** Departamento de Genética Molecular, Instituto de Fisiología Celular, Universidad Nacional Autónoma de México, México City, Mexico

**Keywords:** CTCF, *cis*-regulatory element, enhancer, chromatin, erythropoiesis

## Abstract

**Background:**

*Cis*-regulatory elements (CREs) play crucial roles in regulating gene expression during erythroid cell differentiation. Genome-wide erythroid-specific CREs have not been characterized in chicken erythroid cells, which is an organism model used to study epigenetic regulation during erythropoiesis.

**Methods:**

Analysis of public genome-wide accessibility (ATAC-seq) maps, along with transcription factor (TF) motif analysis, CTCF, and RNA Pol II occupancy, as well as transcriptome analysis in fibroblasts and erythroid HD3 cells, were used to characterize erythroid-specific CREs. An α-globin CRE was identified, and its regulatory activity was validated *in vitro* and *in vivo* by luciferase activity and genome-editing assays in HD3 cells, respectively. Additionally, circular chromosome conformation capture (UMI-4C) assays were used to distinguish its role in structuring the α-globin domain in erythroid chicken cells.

**Results:**

Erythroid-specific CREs displayed occupancy by erythroid TF binding motifs, CTCF, and RNA Pol II, as well as an association with genes involved in hematopoiesis and cell differentiation. An α-globin CRE, referred to as CRE-2, was identified as exhibiting enhancer activity over αD and αA genes *in vitro* and *in vivo*. Induction of terminal erythroid differentiation showed that α-globin CRE-2 is required for the induction of αD and αA. Analysis of TF binding motifs at α-globin CRE-2 shows apparent regulation mediated by GATA-1, YY1, and CTCF binding.

**Conclusion:**

Our findings demonstrate that cell-specific CREs constitute a key mechanism that contributes to the fine-tuning gene regulation of erythroid cell differentiation and provide insights into the annotation and characterization of CREs in chicken cells.

## Introduction

The regulation of gene expression depends on the activity of *cis*-regulatory elements (CREs), such as promoters, enhancers, silencers, and insulators. These regulatory elements have been described as modular sequences with binding sites for transcription factors that participate in a myriad of intricate processes such as development, cell response to stimuli, and cellular differentiation through the regulation of gene expression (Shlyueva et al., 2014; Heinz et al., 2015). Genome-wide studies have helped identify *cis*-regulatory elements through chromatin accessibility, histone modification chromatin marks, transcription factor motifs and binding, and three-dimensional chromatin organization (Hornblad and Remeseiro, 2022). It is interesting to note that although some *cis*-regulatory elements present similar genome-wide characteristics in different cell types, for example, promoters of house-keeping genes, others are active depending on the cell type as is the case for lineage-specific enhancers (Cai et al., 2020). Indeed, it has been shown that the activity of regulatory elements and transcription factors is key for cell differentiation (Herrmann et al., 2022).

The chicken α-globin domain has been historically paradigmatic for the study of gene expression (Beacon and Davie, 2023). Three genes code for the vertebrate-conserved α-globin subunit of hemoglobin in chicken: HBZ (π), HBAD (αD), and HBA1 (αA) (Hardison, 2012). Erythroblast-derived HD3 cells have been adopted as a model to study the molecular mechanisms underlying erythroid cell differentiation *in vitro*, which represents an erythroid cell line transformed by the avian erythroblastosis virus that can be induced to erythroid differentiation by incubating at 42°C in a medium containing protein kinase inhibitor H7 [1-(5-isoquinolinesulfonyl-2-methylpiperazine)] (Nicolas et al., 1991).

In this work, we report a novel *cis*-regulatory element located in the 3ʹ region of the chicken α-globin domain, which is active in the erythroid lineage and characterized by the occupancy of CTCF and histone H3K4me1 and H3K27ac chromatin marks. Furthermore, we showed that CRE-2 is important for the expression of both the adult αD and αA genes in HD3 and differentiated HD3 cells, and that it contacts the promoters of both genes in these cell lines. Thus, we characterized a previously unknown regulatory element that contributes to the understanding of the fine regulation of the expression of the α-globin locus.

## Materials and methods

### Cell culture

Primary cultures of red blood cells (RBCs) were obtained from chicken embryos at 5 and 10 days of development (5dRBCs and 10dRBCs, respectively). The cell culture conditions for RBCs and HD3 cells were as previously described (Rincon-Arano et al., 2009). To induce erythroid differentiation, HD3 cells were treated with H-7 dihydrochloride (20 μM; Sigma) in an HD3 cell medium containing 8% FBS (MultiCell), 2% chicken serum (Gibco), 1% penicillin/streptomycin (Gibco), and 10 mM HEPES, pH 8.0, in a 1% CO_2_ atmosphere at 42°C for 48 h.

### Chromatin immunoprecipitation–qPCR

Chromatin immunoprecipitation (ChIP) for histone modifications (H3K27ac, H3K4me1, and H3K4me3) was performed as described by Valdes-Quezada et al. (2013), with minor modifications. In brief, HD3 cells, HD3-dif cells, 5dRBCs, and 10dRBCs were cross-linked for 10 min in PBS with 1% formaldehyde at a density of 2 × 10^7^ cells/mL, and the cross-linking reaction was quenched for 5 min with 0.125 M glycine. The cells were washed twice with 1× PBS, resuspended in cell lysis buffer (10 mM Tris-HCl [pH 7.5], 10 mM NaCl, and 0.3% NP-40, supplemented with a protease inhibitor cocktail [PIK]), and incubated for 30 min at 4°C. Nuclear fractions were isolated by centrifugation and dissolved in nuclear lysis buffer (50 mM Tris-HCl [pH 7.5], 10 mM EDTA, and 1% SDS and PIK). The chromatin was fragmented by sonication in a bioruptor for 300 s (amplitude 35%) with intervals of 9.9 of pulse on and 9.9 in pause. Chromatin fragmentation was evaluated by agarose gel electrophoresis. For each chromatin immunoprecipitation (IP), 50 µg of chromatin was diluted at 1:5 with dilution buffer (1% Triton X-100, 2 mM EDTA, 20 mM Tris-HCl, and 150 mM NaCl and PIK). The chromatin was precleared with 50 µL of blocked protein G/A beads for 2 h and incubated overnight at 4°C with 2 µg of antibody or IgG. The following antibodies were purchased from Abcam: H3K27ac (#4729), H3K4me1 (#8895), and H3K4me3 (#8580). IgG was purchased from Millipore (#12-371). IPs were carried out by using 30 µL of blocked protein G/A beads for 2 h at 4°C, and the beads were washed as follows: 4× wash buffer I (0.1% SDS, 1% Triton X-100, 2 mM EDTA, 20 mM Tris-HCl, and 150 mM NaCl and PIK) and 1× wash buffer II (0.1% SDS, 1% Triton X-100, 2 mM EDTA, 20 mM Tris-HCl, and 500 mM NaCl and PIK). The chromatin was eluted in elution buffer (1% SDS and 100 mM NaHCO_3_), and a decross-linking reaction was carried out in decross-link buffer (200 mM Tris-HCl, 400 mM NaCl, 0.4% SDS, and 10 mM EDTA) with RNase A (Ambion) for 1 h at 37°C and proteinase K (NEB) for 4 h at 65°C. DNA was purified by adding 1:1 phenol:chloroform:isoamyl alcohol (Invitrogen), mixed by rotation for 10 min at room temperature (RT), and centrifuged for 10 min at 15,294 *g* at RT. Then, DNA was precipitated with 1 M ammonium acetate and glycogen (Roche) in 100% ethanol for 2 h at −70°C, pelleted by centrifugation for 30 min at 17,401 *g*, washed twice with 70% ethanol, and resuspended in 30 µL of nuclease-free water. ChIP-qPCRs contained 1 µL of IP DNA or 1% input and were performed using iTaq Universal SYBR Green Supermix (Bio-Rad). The oligonucleotides used for ChIP-qPCR are listed in Supplementary Table S1.

### Luciferase reporter assay

The CRE-2 DNA sequence (chr14:12512783–12514878, *Gallus gallus*: GRCg6a/galGal6) was amplified by PCR from HD3 genomic DNA and cloned downstream of the luciferase gene into the BamHI site of the pGL3-Basic vector (Promega). For π, αD, and αA, promoter sequences were amplified from HD3 genomic DNA by PCR and cloned upstream of the luciferase gene at the DpnII site of pGL3-Basic (control) or pGL3-CRE-2. Erythrocytes isolated from 10-day-old embryos (10d RBC) were used for cell transfection. A measure of 5 × 10^5^ 10dRBCs were cotransfected with 1 µg of each plasmid and 200 ng of *Renilla* vector using the Lipofectamine 2000 Reagent (Invitrogen). Luciferase activity was measured 24 h after transfection using the Dual-Luciferase Reporter Assay kit (Promega) on a Luminometer TD-20 (Turner Designs). Luciferase values were normalized to *Renilla* luciferase values. Relative luciferase units of each promoter construct containing CRE-2 were determined as fold change with respect to the pGL3-Promoter (control without CRE-2). The oligonucleotides used for luciferase reporter assay are listed in Supplementary Table S1.

### Circular chromosome conformation capture with the unique molecular identifier protocol

The circular chromosome conformation capture with the unique molecular identifier (UMI-4C) protocol was performed as described by Schwartzman et al. (2016) with minor modifications. Two replicates of experiments were performed for each condition (HD3 and HD3-dif cells). In brief, a total of 2 × 10^7^ cells were centrifuged at 239 *g* for 5 min in a 15-mL Falcon tube. We discarded the supernatant, resuspended the pellet in 1 mL of PBS, and transferred it to a 2-mL Eppendorf tube. The cells were washed out of the PBS, and cross-linking was performed with 1 mL of PBS–1% paraformaldehyde for 10 min at RT with rotation. The sample was quenched with glycine for 5 min at a final concentration of 0.125 M on ice. The cells were washed with PBS at 4°C, and the pellet was stored at −80°C. For nucleus extraction, fixed cells were homogenized in 1 mL of freshly prepared cell lysis buffer (50 mM Tris, pH 7.5; 150 mM NaCl; 5 mM EDTA; 0.5% NP-40; 1.15% Triton X-100; 1× Roche cOmplete protease inhibitors) and incubated on ice for at least 10 min. The nuclei were pelleted by centrifugation for 5 min, 750 *g* at 4°C, and washed with 1× PBS. The nucleus pellets were resuspended in 100 µL 0.5% SDS and incubated for 10 min at 62°C without shaking. The samples were moved to ice, and 292 µL water and 50 µL 10% Triton X-100 were added to each sample, mixed, and incubated for 15 min at 37°C to quench the remaining SDS. The chromatin was digested with 400 units of DpnII (NEB, R0543) in 50 µL of 10× restriction enzyme (RE) buffer at 37°C for 4 h with 86 *g* shaking, and then, 400 units more of DpnII were added plus 450 µL of 1× RE buffer for overnight incubation under the same conditions. DpnII was then heat-inactivated at 65°C for 20 min. Biotin fill-in and proximity ligation was performed with Klenow (NEB, M0210L) and T4 DNA ligase (NEB, M0202L), respectively, as described by Franke et al. (2021). The nucleus pellet was centrifuged at 600 *g* for 10 min at 4°C, 800 µL of the supernatant was removed, and 230 µL of proteinase K buffer, 20 µL of proteinase K (10 mg/mL), and 50 µL of 10% SDS were added for incubation at 55°C for 30 min. Then, 40 µL of 4 M NaCl was added, and incubation continued at 65°C overnight with 52 *g* shaking. The next day, 5 µL of RNAse A (10 mg/mL) was added, followed by incubation at 37°C for 30 min at 52 *g*. A measure of 20 μL of proteinase K (10 mg/mL) was added to the sample and incubated at 55°C for 1–2 h at 52 *g*. DNA was purified by phenol–chloroform extraction and precipitated with NaAc, glycogen, and cold 100% ethanol. The precipitated DNA was eluted in 100 µL of 10 mM Tris-HCl, pH 7.5. Subsequently, 7 µg of purified DNA was sheared using a Covaris M220 sonicator, and the samples were purified using AMPure XP beads (Agencourt, A63881). DNA was resuspended in 300 µL water. Biotin-labeled DNA was bound to Dynabeads MyOne Streptavidin C1 beads using 5 µL of beads per 1 µg DNA by following the manufacturer’s instructions to perform the pull-down process. Reclaimed beads were eluted in 50 µL water. A measure of 500 ng of DNA was attached to the streptavidin beads following sequential incubation using end-repair mix (1× T4 Ligase Buffer [NEB], 0.5 mM dNTP mix, 0.12 U/µL T4 DNA Polymerase [NEB, M0203], and 0.05 U/µL Klenow [NEB, M0210]), A-tailing mix (1× NEB buffer 2, 0.5 mM dATP, and 0.25 U/µL Klenow, exo- [NEB, M0212]), and CIP (NEB, M0290), as described by Franke et al. (2021). The samples were indexed by ligating TruSeq Illumina adapters by incubating DNA-bound beads in an adapter ligation mix (1× T4 Ligation Buffer, 5% PEG-4000, and 0.3 U/µL T4 DNA ligase (Thermo Fisher, EL0011)) at RT for 2 h. The DNA-bound beads were denatured to remove the non-ligated strand of the adaptor. After washing, the DNA-bound beads were resuspended in 20 µL water. Then, 200 ng of DNA-bound beads were used for library preparation by nested PCR. Three PCRs (20 cycles) were performed and then pooled for AMPure bead purification for each sample for the first PCR. In addition, for the second PCR (10 cycles), 4 PCRs were performed for each sample and then pooled for size selection for fragments between 200 and 700 bp using AMPure beads. Libraries were multiplexed and sequenced using DNBSEQ technology to produce 50 bp paired-end reads and approximately 1–4 million raw sequencing read pairs for the CRE-2 viewpoint for each sample. For the UMI-4C data analysis, raw FASTQ files were processed using UMI4Cats 1.12.0 of the R package (https://github.com/Pasquali-lab/UMI4Cats). For the visualization of the chromatin contact profiles, we used the default parameters (Ramos-Rodriguez et al., 2021). The oligonucleotides used for UMI-4C are listed in Supplementary Table S1.

### CRISPR–Cas9 genome editing

For CRISPR-based experiments, the two single-guide RNAs (sgRNAs) used in this study were designed using the CRISPOR web tool (http://crispor.gi.ucsc.edu/) (Concordet and Haeussler, 2018) using the galGal6 genome annotation. sgRNAs with high specificity scores (>70), as recommended by the CRISPOR tools, were purchased from Sigma. Oligonucleotides for the guides were cloned into pSpCas9(BB)-2A-Puro (PX459) V2.0 (provided by Feng Zhang, Addgene #62988), as described by Ran et al. (2013). The integrity of the cloned guides was evaluated by Sanger sequencing. The plasmids were expanded and purified from the top 10 *E. coli* competent cells. A measure of 500 ng of each plasmid was transfected together into 5 × 10^5^ HD3 cells with the Lipofectamine 2000 Reagent (Invitrogen) according to the manufacturer’s guidelines. A measure of 1 μg/mL of puromycin (Sigma) was added to the transfected cell culture after 24 h of plasmid transfection. After 7 days of puromycin selection, an aliquot of cells was used for DNA extraction by phenol–chloroform and PCR genotypification using specific primers spanning the desired deletion. The pool of HD3 mutant cells was used for serial dilutions from 1 × 10^6^ cells/mL until 5 cells/mL was reached in the HD3 medium, and 100 µL of the last dilution was deposited per well in a 96-well plate. Single clones were identified by microscopy after 2 weeks and expanded for subsequent genotypification by PCR. The deletions for both mutant cell clones were further characterized by cloning the PCR fragments obtained from genotypification into the pGEM-T Easy vector (Promega) and confirmed by Sanger sequencing. The oligonucleotides used for CRISPR-Cas9 genome editing assay are listed in Supplementary Table S1.

### Analysis of gene expression by RT-qPCR

Total RNA was isolated using TRIzol Reagent (Invitrogen) according to the manufacturer’s instructions, with minor modifications. The HD3 cells were pelleted and resuspended in TRIzol Reagent. RNA was extracted using phenol–chloroform, washed twice with 75% ethanol, and resuspended in nuclease-free water. A measure of 50 ng of RNA was used in each RT-qPCR to determine the target abundance using the KAPA SYBR FAST One-Step Kit (KAPA Biosystems), using the StepOne Real-Time PCR System. RPL27 was used as an endogenous control. RT-qPCR data were analyzed by the ΔΔCt method (Livak and Schmittgen, 2001). The oligonucleotides used for RT-qPCR are listed in Supplementary Table S1.

### ATAC-seq data analysis

Public ATAC-seq datasets used in this study were retrieved from GSE206194. Raw reads were aligned to the chicken genome assembly galGal6 (GRCg6a) using Bowtie 2 with default parameters (Langmead et al., 2009). Properly paired reads were included; reads marked as secondary alignments, PCR duplicates, and low-quality mapped reads were removed using NGSUtils (Breese and Liu, 2013). Filtered bam files from each replicate were merged and used for peak calling using MACS2 using an FDR <0.01 (Zhang et al., 2008). Overlapped (conserved) ATAC peaks between HD3 cells and fibroblasts were identified using BEDTools (Quinlan and Hall, 2010). Plot profiles and heatmaps of ATAC-seq density over given regions were generated using deepTools2 using bigwig files obtained from bam files normalized as counts per million (CPM) mapped reads (Ramirez et al., 2016). ATAC peaks were annotated using ChIPseeker (Yu et al., 2015).

### ChIP-seq data analysis

Public ChIP-seq datasets for RNA POL II (GSE228694) and CTCF (GSE51846) were retrieved from the European Nucleotide Archive (ENA). Raw reads were aligned to the chicken genome assembly galGal6 (GRCg6a) using Bowtie 2 with default parameters (Langmead et al., 2009). Properly paired reads were included; reads marked as secondary alignments, PCR duplicates, and low-quality mapped reads were removed using NGSUtils (Breese and Liu, 2013). Filtered bam files from each replicate were merged and used to generate normalized read-density files as CPM using deepTools2 (Ramirez et al., 2016).

### RNA-seq data analysis

Public RNA-seq datasets used in this study were retrieved as follows: fibroblasts and HD3 cells (GSE206193) and non-induced HD3 and induced HD3 cells (GSE76573). Raw reads were mapped to chicken genome assembly galGal6 (GRCg6a) using STAR with default parameters (Dobin et al., 2013). Gene counts were assessed using featureCounts with NCBI RefSeq annotation release 104 (Liao et al., 2014). Differential gene expression analysis was performed using edgeR (Robinson et al., 2010). Differentially expressed genes were identified by filtering in R as follows: FDR <0.05 and log2FC ± 0.5. Mapped reads of each replicate were merged and filtered as follows: properly paired reads were included; reads marked as secondary alignments, PCR duplicates, and low-quality mapped reads were removed using NGSUtils (Breese and Liu, 2013). Read-density files were generated from the filtered mapped reads using deepTools2 (Ramirez et al., 2016). Normalized counts as CPM from edgeR were used for heatmap visualization.

### Genome-wide motif discovery

Motif analysis of ATAC peaks was performed using *findMotifsGenome.pl* from HOMER using vertebrate motif collection, GC% sequence normalization, and *p*-values determined by the hypergeometric test (Heinz et al., 2010). The top eight highly significant transcription factor (TF) motifs by *p*-value and FDR < 1e-10 of each group of ATAC peaks were used for visualization.

### Gene Ontology analysis

The biological processes (GO terms) of gene sets were conducted using Metascape using multiple gene lists containing annotated genes for each group (genic, intergenic, and intragenic) (Zhou et al., 2019). Gene Ontology (GO) terms were filtered out by the Benjamini–Hochberg test with an FDR < 1e-10. The top 20 highly significant GO terms by *p*-value (binomial distribution) were selected for visualization as a heatmap.

### Transcription factor binding motif analysis

We performed a binding motif search using FIMO (*p*-value < 1e-5) (https://meme-suite.org/meme/tools/fimo) (Grant et al., 2011) of the DNA sequence comprehended between the two sgRNAs. We used the matrix of core binding sites for vertebrates’ redundant transcription factors of MEME found in the JASPAR website (https://jaspar.elixir.no/downloads/) (Rauluseviciute et al., 2024). From the obtained redundant binding motifs, we excluded from the analysis the binding motifs that had overlapped sequences for the same transcription factor. We looked for the chicken genes orthologous to the human transcription factor binding motifs identified by FIMO using the database of the Orthologous Matrix (OMA) browser (https://omabrowser.org/oma/genomePW/) (Altenhoff et al., 2021) with its option of Genome Pair View (species 1: human; species 2: chicken; preferred ID: source data I). Then, we examined the overall transcription levels of the candidate chicken transcription factors in the HD3 cells and compared their expression against fibroblasts (Penagos-Puig et al., 2023) and HD3-dif cells (Ulianov et al., 2017).

### Statistical analysis

Data are represented as the mean ± standard error of two biological replicates or are otherwise specified in the figure legends. *p*-values were determined by the unpaired Student’s t-test with Welch’s correction using GraphPad Prism9.

## Results

### Identification of chicken erythroid-specific regulatory elements

**FIGURE 1:**
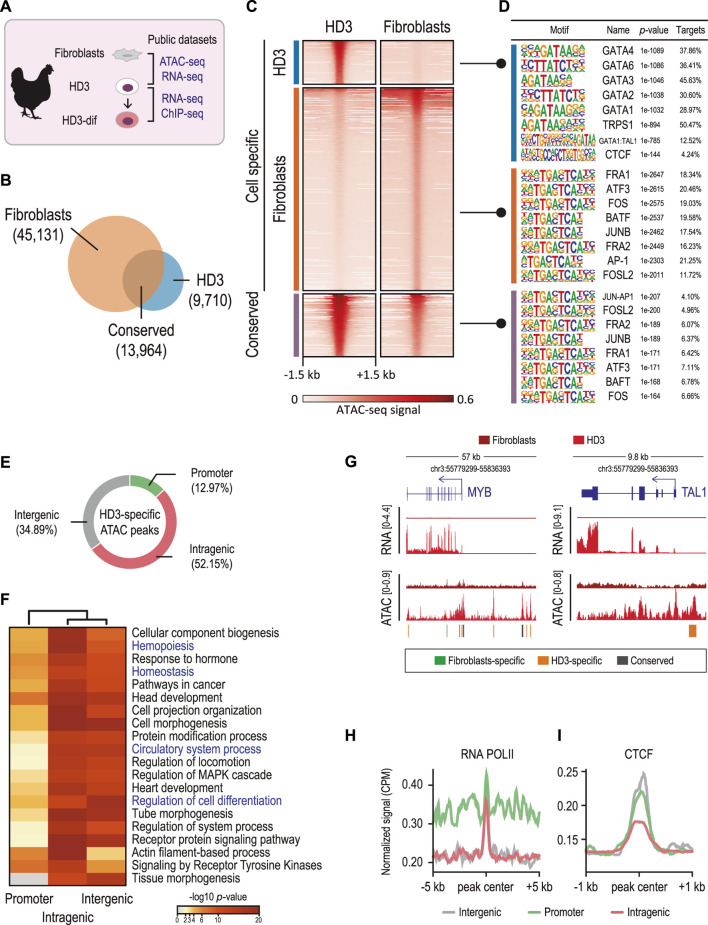
Genome-wide accessibility analysis reveals *cis*-regulatory elements (CREs) in chicken erythroid cells. **(A)** Public high-throughput sequencing data used in this study for chicken fibroblasts (ATAC-seq and RNA-seq), HD3 (ATAC-seq, ChIP-seq, and RNA-seq), and HD3-dif cells (RNA-seq) were obtained from the studies by Penagos-Puig et al. (2023); Ulianov et al. (2017); Gushchanskaya, et al. (2014). **(B)** Venn diagram showing the overlapped (conserved) and cell-specific ATAC peaks. **(C)** Heatmaps of normalized ATAC-seq signals at the identified ATAC peaks. **(D)** Top eight TF binding motifs identified in each set of ATAC peaks. *p*-values were determined by a hypergeometric test. **(E)** ATAC peak distribution using the nearest neighbor gene approach. ATAC peaks overlapping ± 1 kb from the annotated TSS were assigned as promoters; peaks overlapping exons, introns, and URTs were annotated as intragenic; and peaks non-overlapping annotated genes were designed as intergenic. **(F)** Heatmap showing the significance (*p-*values) of the annotated biological process (GO terms) for each set of genes associated with ATAC peaks. *p*-values were determined by a binomial distribution test. **(G)** Genomic regions showing RNA-seq and ATAC-seq signals corresponding to *MYB* and *TAL1* genes. Conserved (gray) and cell-specific peaks (green and orange) are shown. **(H,I)** Plot profiles of normalized ATAC and CTCF ChIP-seq signals at the annotated ATAC peaks.

To systematically explore chromatin accessibility underlying the functional specificity of regulatory elements that control cell-specific gene expression in chicken cells, we conducted integrative analyses of fibroblast and proerythroblast (HD3) cells. Specifically, we analyzed public data for genome accessibility by ATAC-seq and transcriptomic profiles by RNA-seq (Figure 1A) (Ulianov et al., 2017; Penagos-Puig et al., 2023). We identified cell-specific (n = 9,710 in HD3 cells and n = 45,131 in fibroblasts) and conserved (n = 13,964 in HD3 cells and fibroblasts) regions of chromatin accessibility (Figures 1B, C). These cell-specific chromatin-accessible regions likely represent CREs in chicken cells. We then performed motif analyses to identify TF binding motifs in these accessible regions. Common binding motifs for ubiquitous transcription factors, such as FRA, ATF3, JUNB, and BAFT, were detected in both conserved and fibroblast-specific ATAC peaks, whereas the HD3-specific chromatin-accessible regions include binding motifs for the GATA family members, which are well-known hematopoietic and erythroid regulators (Figure 1D). The enrichment of GATA binding motifs in HD3-specific ATAC peaks validates the erythroid nature of these potential CREs. Notably, the binding motif for the chromatin architectural protein CTCF was also enriched in HD3-specific ATAC peaks, supporting the role of chromatin architecture in the regulation of cell differentiation. Altogether, these findings are consistent with the observed combinatorial activity of transcription factor binding on CREs, which are important determinants in driving cell-specific gene expression (Huang et al., 2016; Georgolopoulos et al., 2021; Ranzoni et al., 2021).

To explore whether HD3-specific ATAC peaks were associated with erythroid cell identity genes, we assigned HD3-specific ATAC peaks to proximal genes (Figure 1E). We found that HD3-specific ATAC peaks were associated with genes involved in hemopoiesis, hemostasis, and circulatory system processes (Figure 1F). Notably, genes associated with intergenic HD3-specific ATAC peaks were significantly enriched in cell differentiation (*p-*value = 4.046e-19). For example, the key transcription factor regulators of vertebrate hematopoiesis MYB and TAL1 were highly expressed in HD3 cells but not in fibroblasts, which is consistent with observed genes associated with cell differentiation and hematopoiesis containing HD3-specific intergenic ATAC peaks (Figure 1G). These data suggest that HD3-specific ATAC peaks represent HD3-specific CREs involved in controlling the transcription of hematopoietic genes.

Given that HD3-specific ATAC peaks were enriched in intragenic and intergenic regions, these peaks might represent distal regulatory elements, such as enhancer elements, which often are bound with RNA polymerase II (RNAPII) and architectural proteins, such as CTCF (Kubo et al., 2021; Larke et al., 2021). Analyzing ChIP-seq data showed that HD3-specific ATAC peaks were enriched for RNAPII (Figure 1H), whereas intergenic and promoters were highly occupied by CTCF (Figure 1I). These results suggest that CTCF and RNAPII occupancy between intergenic and promoter CREs might cooperate to control the expression of hematopoietic and cell differentiation genes, which has been extensively described in previously reported roles of enhancer–promoter interactions in driving hematopoietic and erythroid cell differentiation in vertebrates (Chen et al., 2019; Edginton-White et al., 2021; Kim et al., 2023).

### A CRE embedded into the α-globin domain is associated with αD and αA activation during terminal erythroid differentiation

**FIGURE 2:**
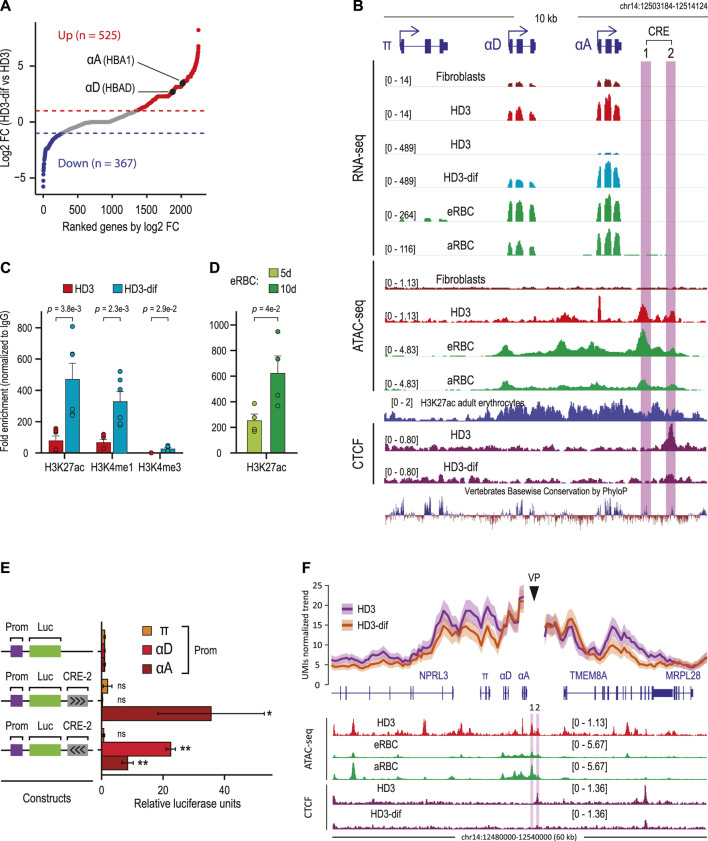
The chicken α-globin domain contains an erythroid-specific CRE associated with αD and αA activation. **(A)** Scatter plot showing differentially expressed genes ranked by fold change gene expression (log2FC) between HD3 and induced HD3 (HD3-dif) cells. Significantly upregulated (FDR <0.05, log2FC > 0.5) and downregulated (FDR <0.05, log2FC < −0.5) genes are shown in red and blue, respectively. **(B)** α-Globin domain containing the embryonic (π) and adult (αD and αA) genes. Normalized RNA-seq, ATAC-seq, and ChIP-seq data are shown. Conserved DNA sequences between vertebrates are shown by PhyloP track from the UCSC Genome Browser. CRE-1 and CRE-2 genomic regions are highlighted in purple. **(C)** ChIP-qPCR for active (H3K27ac) promoter (H3K4me3) and enhancer (H3K4me1) histone marks overlapping the CRE-2 genomic region in HD3 and HD3-dif cells. *p*-values were calculated by Student’s t-test for two independent experiments. **(D)** ChIP-qPCR for the active (H3K27ac) histone mark overlapping the CRE-2 genomic region in erythrocytes from 5 (5d) and 10 (10d) days of developmental embryos. *p*-values were calculated by Student’s t-test for two independent experiments. **(E)** Luciferase reporter assays of CRE-2 activity over α-globin genes. A 2.089-kb fragment containing the CRE-2 DNA sequence was cloned into pGL3-Basic coupled with a single promoter (π, αD, and αA). Black arrows indicate the orientation of the CRE-2 sequence. *p*-values were calculated by Student’s t-test for three independent experiments and summarized as follows: not significant (ns), *p < 0.05, and **p < 0.01. **(F)** (Top) UMI-4C assay in non-induced (HD3) and induced (HD3-dif) cells using the CRE-2 genomic region as the viewpoint (VP). Lines represent the average normalized UMI counts, and their standard deviation is shown in shadows. (Bottom) Normalized read signals for ATAC in erythroid cells (HD3 cell, eRBC, and aRBC) and CTCF ChIP-seq in HD3 cells.

To explore whether HD3-specific CREs drive the expression of erythroid-specific genes, we focused on intergenic HD3-specific CREs for two reasons: first, these associate with genes involved in erythroid differentiation, and second, these are occupied by the RNA POL II and CTCF, which is consistent with the previously reported functions of intergenic enhancers in regulating hematopoietic specification and differentiation (Edginton-White et al., 2021). We analyzed public RNA-seq data from HD3 and HD3 cells induced to *in vitro* erythroid differentiation and identified differentially expressed genes associated with intergenic CREs (Figure 2A) (Ulianov et al., 2017). Notably, by focusing on upregulated genes, we observed that adult α-globin genes (αD and αA) were significantly upregulated according to erythroid maturation (Gasaryan, 1982). Interestingly, two CREs (CRE-1 and CRE-2) downstream from αD and αA genes were identified. By analyzing public ATAC-seq and RNA-seq data, we also observed that both CREs were accessible in embryonic (eRBC) and adult (aRBC) chicken erythrocytes, which coincides with the abundant gene expression of αD and αA but not with the embryonic α-globin gene π (Figure 2B). Importantly, both CREs showed conserved DNA sequences, suggesting that these elements might exert conserved activity on α-globin genes in vertebrates (Fish et al., 2017). These analyses indicate that HD3-specific intergenic CREs correlate with pronounced transcriptional changes during terminal erythroid differentiation.

CREs, such as enhancers, can exert critical regulatory activities during erythroid cell specification and development (Huang et al., 2016; Schulz et al., 2019; Georgolopoulos et al., 2021). The CRE-1 region has been previously described as an enhancer regulated by GATA-1 and YY1 (Rincon-Arano et al., 2005), and interestingly, CRE-2 is bound by CTCF, according to ChIP-seq public data on HD3 cells. We hypothesized that CRE-2 might regulate αD and αA transcriptional activity in erythroid cells. To directly explore this, we analyzed enhancer-associated histone modifications in HD3 cells non-induced and induced to differentiate. We observed that in HD3 cells, CRE-2 was marked by active enhancer-related histone modifications H3K27ac and H3K4me1, which significantly increased upon differentiation (HD3-dif) (Figure 2C). Similarly, H3K27ac was present in CRE-2 in erythrocytes isolated from 5-day and further enriched in 10-day developed chicken embryos, indicating that CRE-2 activity is dynamic during erythroid differentiation and development.

The chromatin profile of CRE-2 suggests that this may be an enhancer element. Therefore, we evaluated the enhancer activity of CRE-2 on α-globin gene promoters by luciferase reporter assays. We inserted the CRE-2 DNA sequence, corresponding to ∼2.6 kb, including the CTCF-binding site and the occupied H3K27ac region, in both forward and reverse orientations downstream of the luciferase gene driven by π, αD, and αA promoters in 10-day-old RBCs. Notably, CRE-2 transactivates only the adult αD and αA promoters in both orientations, as measured by luciferase activity (Figure 2E).

Enhancer elements control spatiotemporal gene expression during a broad range of biological processes, including erythroid cell differentiation, through physical proximity to their cognate gene promoters (Uyehara and Apostolou, 2023). To directly explore the spatial proximity of CRE-2 alongside α-globin genes during erythroid differentiation and to investigate the CRE-2 chromatin interactions with high resolution, we performed UMI-4C using this regulatory element as a viewpoint (VP) (Schwartzman et al., 2016). We found that in HD3 cells, the contacts from the CRE-2 region spanned approximately 50 kb, including the α-globin genes and other non-globin genes (NPRL3 and TMEM8), suggesting that the α-globin domain is structured so that CRE-2 brings into proximity NPRL3, π, αD, αA, and TMEM8A before gene activation (Figure 2F). In contrast, chromatin contacts within the α-globin locus were reduced around the VP in HD3-dif cells, including NPRL3, π, αD, and TMEM8A, while short-range contacts at αA were maintained. These data indicate that CRE-2 is part of the α-globin regulatory landscape with an apparent regulatory and structural function.

CTCF binding is essential to mediate enhancer–promoter interactions during cell differentiation (Kubo et al., 2021; Qi et al., 2021; Song et al., 2022). Interestingly, analysis of public CTCF ChIP-seq data showed that CTCF binding was decreased at the CRE-2 site and at TMEM8A (Figure 2F, cherry tracks) (Gushchanskaya et al., 2014). This suggests that the decreased chromatin interactions between CRE-2 and α-globin genes might occur as a consequence of decreased CTCF binding in HD3-dif cells. In addition, these data indicate that CTCF and CRE-2 might cooperate to organize the α-globin domain and facilitate gene activation in HD3-dif cells.

### CRE-2 is required for proper αD and αA transcriptional activity in erythroid cells

**FIGURE 3:**
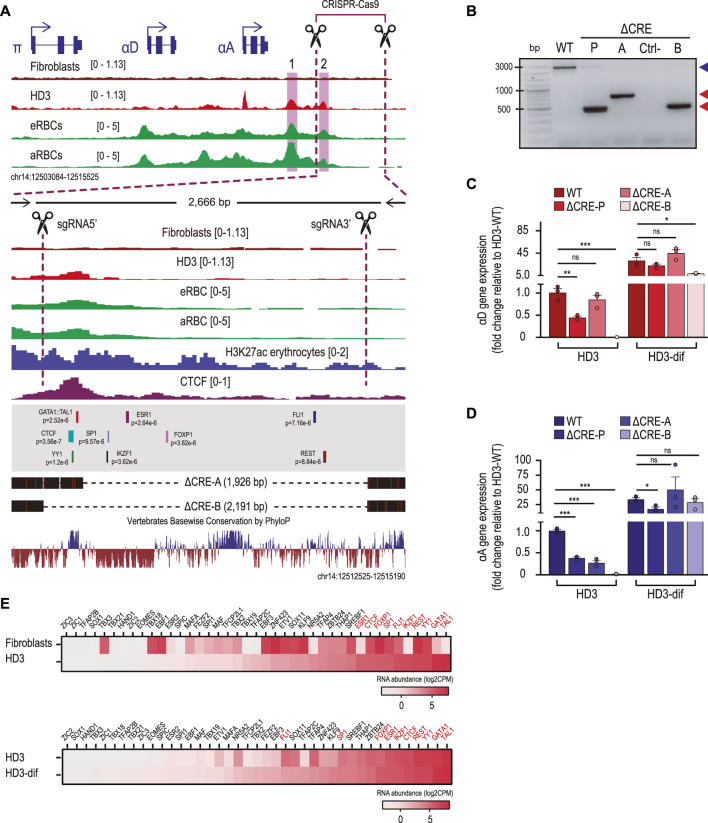
The CRE-2 region is required for the induction of αD and αA gene expression. **(A)** Normalized ATAC-seq and CTCF ChIP-seq regions of the α-globin domain. The dotted lines represent a close-up view of the deleted region by the CRISPR–Cas9 system. The black dotted lines represent the deleted regions (1,926 and 2,191 bp) corresponding to the cell clones ΔCRE-A and ΔCRE-B, respectively, obtained by PCR screening and Sanger sequencing. Overlapped TF binding motifs identified using FIMO and conserved genomic sequence by PhyloP are shown. The black arrows represent the oligonucleotides used for PCR screening, and the black scissors represent the positions of the single RNAs (sgRNAs) used for CRISPR–Cas9-mediated deletions. **(B)** Agarose gel showing products of PCR genotypification from non-edited (WT) cells, the pool of cells after puromycin selection (ΔCRE-P), and two cell clones (ΔCRE-A and ΔCRE-B) obtained by puromycin selection and isolated by cloning dilution. The blue arrow represents the non-edited genomic region (2,600 bp), and the red arrows correspond to ∼500 bp of the deleted cell clones ΔCRE-A and ΔCRE-B. **(C, D)** Gene expression of αD and αA determined by RT-qPCR in non-induced (HD3) and induced (HD3-dif) cells for non-edited (WT) and edited (ΔCRE-P, ΔCRE-A, and ΔCRE-B) cells. Gene expression was normalized to the non-induced (HD3) condition (WT). The bar charts show the mean ± SD of two independent experiments. *p-*values were determined by Student’s t-test and summarized as follows: not significant (ns), *p < 0.05, **p < 0.01, and ***p < 0.001. **(E)** Heatmap of the transcription factor abundance for fibroblasts and erythroid cells (HD3 and HD3-dif) obtained from RNA-seq data analysis. RNA abundance is shown as log2 CPM.

To assess whether CRE-2 directly contributes to the regulation of α-globin gene expression, we designed two gRNAs to direct the Cas9 endonuclease and remove a region of approximately 2.2 kb in HD3 cells, spanning CRE-2 and the downstream sequence, which is highly conserved among vertebrates (Figure 3A). We obtained a pool of edited cells showing the expected deletion (ΔCRE-P) and derived two different clones lacking 1,926 bp (ΔCRE-A) and 2,191 bp (ΔCRE-B), respectively (Figure 3B). We then determined the expression levels of αD (Figure 3C) and αA (Figure 3D) in the edited cells and after the induction of differentiation. In the pool, the levels of both α-globin transcripts decrease by half compared to the control. In addition, although the genes are transcriptionally activated upon differentiation, these do not reach WT levels. Similar results were observed in the ΔCRE-B clone, in which both α-globin transcripts were downregulated in non-induced cells and remained at lower levels upon induction to differentiation. In contrast, the ΔCRE-A clone retains αD expression similar to the control in both non-induced and induced cells, while αA transcript levels are restored upon differentiation. Interestingly, an important sequence encompassing CRE-2 is retained in the ΔCRE-A clone, which is lost in the ΔCRE-B cells and in most of the edited cells from the pool (Figures 3A, B). This prompted us to perform a binding motif search using FIMO (*p*-value <0.00001) in the whole DNA sequence flanked by the two gRNAs to explore whether the absence of a subset of transcription factors could explain the misregulation of the α-globin genes in the edited cells and whether some of these transcription factors are retained in the ΔCRE-A cells. We obtained 87 redundant binding motifs and excluded those that had overlapped sequences for the same transcription factor, ending with 57 binding motifs of 54 different transcription factors. We identified 41 chicken orthologs of the 54 human transcription factors, according to the human Ensembl gene annotation. Then, we examined the overall expression levels of these factors in HD3 cells compared to fibroblasts and HD3-dif cells. The top 10 included erythroid-specific transcription factors (such as the orthologs of TAL1, GATA1, IKZF1, and ESR1), as well as constitutive regulators (such as the orthologs of YY1, REST, FLI1, SP1, FOXP1, and CTCF) (Figure 3E). The predicted binding sites of the top 10 expressed transcription factors in HD3 cells are mapped in Figure 3A. As shown, ΔCRE-A cells preserve the binding motifs for TAL1, GATA1, CTCF, and YY1. This analysis suggests that intact CTCF, YY1, and GATA-1:TAL1 binding motifs at CRE-2 are necessary to facilitate the full activation of αD and αA in erythroid cells.

## Discussion

In this work, we identified active regulatory elements in chicken erythroid cells. We found a ∼2.2-kb region downstream of the α-globin genes of the open chromatin, as shown by ATAC-seq data. This region is specific to the erythroblast HD3 cell line as compared to chicken fibroblasts. To assess its activity as a potential *cis*-regulatory element, we deleted it and found that it had an effect on the expression of the αD and αA genes in both HD3 and HD3-differentiated cells. We demonstrated that the element interacts with the promoters of both αD and αA, which may account for the decrease in their expression levels. It has been described that enhancer–promoter contacts can precede the activation of the expression of a gene as if they are priming it. The regulation of gene expression is mediated by *cis*-regulatory elements. The element that we discovered presents the canonical enhancer mark H3K4me1, the absence of the promoter-associated H3K4me3, and the H3K27ac mark associated with active enhancers (Creyghton et al., 2010). We showed that this region has TFBS for TFs such as GATA1, which is a master regulator of erythroid differentiation and binds with chicken erythroid regulatory elements (Rincon-Arano et al., 2005). Interestingly, the region has a predicted CTCF TFBS that corresponds to a ChIP-seq peak deleted only in the ΔCRE-B cell, which presents the lowest levels of αD and αA expression compared to HD3, ΔCRE-A, and ΔCRE-P cells. CTCF binds to *cis*-regulatory elements and participates in loop formation (Hsieh et al., 2022). Of note, ΔCRE-A presents similar expression levels of αA to HD3 cells upon differentiation, suggesting that αA expression regulation is mediated by other factors besides CRE-2. However, ΔCRE-A does affect the levels of αD also when the cells are differentiated. This shows that the chicken αD and αA genes have different mechanisms of gene regulation. Because of the results previously described, we suggest that CRE-2 contains an enhancer element. It will be of interest to finely elucidate this element through shorter deletions, point mutations, and reporter gene assays. Overall, our results show the discovery and characterization of a CRE region in the chicken α-globin domain previously not explored. This contributes to the understanding of the gene regulation of a cell type-specific multi-gene locus.

## Data Availability

The original contributions presented in the study are included in the article/Supplementary Material; further inquiries can be directed to the corresponding author.
