# Supplementary material for: A novel cis-regulatory element regulates αD and αA-globin gene expression in chicken erythroid cells
Source: Front Genet. 2024 Apr 19;15:1384167. doi: 10.3389/fgene.2024.1384167 (PMC11066237; doi:10.3389/fgene.2024.1384167)
Supplement: Supplementary file 1 [file Table1.pdf]

SUPPLEMENTARY MATERIAL

Supplementary Table 1

Name	Sequence (forward)	Sequence (reverse)	Use
**Enh_Scree**	GATCTGGTCTGCTCCAAACCCT	GGCAGGGAGGGAGAATGAACTT	PCR
**sgCRE-2_1**	CACCGGGGATGATGTGTGCAAGGTT	AAACAACCTTGCACACATCATCCC	sgRNA
**sgCRE-2_2**	CACCGGACCTGCAGTAGTTTGACGT	AAACACGTCAAACTACTGCAGGTC	sgRNA
**RPL27**	AGTTTATGAAGCCGGGGAAGGT	TGCTGTAACCTTCCGTGGGTAG	RT-qPCR
**HBAD (αD)**	ACTGCCGAGGACAAGAAGCTCA	GGTCGAAGTGGGGGAAGTAGGT	RT-qPCR
**HBA1 (αA)**	CACGGCAAGAAGGTAGTGGCTG	CACCACCACCAGGAAGCATTGG	RT-qPCR
**NPRL3**	CTTACGGGCCAATGCAGATC	CCTTCTGTAGTCTCTGACATG	RT-qPCR
**TMEM8**	AGCTGCACAGACGATACCA	TTCAGAATCTTCTTCACCC	RT-qPCR
**CRE-2_ChIP**	CACCTCACTGCCCTCTATGTGA	AGGATGCTTGCCTTCTCTTGTCT	ChIP-qPCR
**Pi_prom**	CGGGATCCGCTCAAAATCCATTGAAAGGCACG	CGGGATCCCCAGAGCAGGTTGTACTGAG	Luciferase reporter
**aD_prom**	CGGGATCCCCTGCATGCAGTGTGGAGC	CGGGATCCGGGCTGGTGGCTGGTGG	Luciferase reporter
**aA_prom**	CGGGATCCCTCCACCTGCACCAAGGCAGACC	CGGGATCCGTGCCCCTGAAGTCTGCTGTCAGC	Luciferase reporter
**CRE-2_luc**	GCGTGGGATCCCAGTTGCTCTCACACCTTGC	GCGTGGGATCCGGTTCTGCCATGGGTATCTCC	Luciferase reporter
**CRE-2**	UMI4C_US	CTTCAATCAGTCGCTGAGGTA	UMI-4C
**CRE-2**	UMI4C_DS	TTGAACTGTACTTTAATCCACTCCT	UMI-4C
